# Methylation changes and *INS-IGF2* expression predict progression in early-stage Wilms tumor

**DOI:** 10.1186/s13148-024-01775-y

**Published:** 2024-11-26

**Authors:** Deena Jalal, Mohamed Y. Ali, Naglaa Elkinaai, Abdelaziz S. Abdelaziz, Wael Zekri, Ahmed A. Sayed

**Affiliations:** 1 Genomics and Epigenomics Program, Department of Basic Research, Children’s Cancer Hospital Egypt, Cairo, 57357 Egypt; 2 Department of Pathology, Children’s Cancer Hospital Egypt, Cairo, 57357 Egypt; 3 Department of Pathology, National Cancer Institute, Cairo University, Cairo, Egypt; 4 Department of Pediatric Oncology, Children’s Cancer Hospital Egypt, Cairo, 57357 Egypt; 5 Department of Pediatric Oncology, National Cancer Institute, Cairo University, Cairo, Egypt; 6 Department of Biochemistry, Faculty of Science, Ain Shams University, Cairo, Egypt

**Keywords:** Wilms tumor, DNA methylation, Epigenetic biomarkers, *INS-IGF2* transcript

## Abstract

Wilms tumor, the most common pediatric kidney cancer, accounts for 5% of childhood cancers and is classified by stage and histological subtype. Despite high survival rates (80–85%), approximately 15% of patients experience relapse, reducing survival to around 50%. Epigenetic changes, particularly DNA methylation, play a critical role in Wilms tumor pathogenesis. This study investigates the prognostic potential of DNA methylation in stage I and II patients with favorable histology, aiming to identify early relapse biomarkers. Genome-wide methylation was assessed using methylation microarrays in tumor tissues from relapsed patients (*n* = 9) and those with complete responses (*n* = 9), alongside normal tissues (*n* = 3 each). Differentially methylated probes and regions were analyzed, with additional ROC and survival analyses. Real-time PCR was used to measure *IGF2* and *INS-IGF2* gene expression. The analysis revealed hypomethylation in intergenic regions in remission patients, identifying 14 differentially methylated positions as potential biomarkers. Increased *INS-IGF2* expression was associated with relapse, suggesting its role in disease progression. While the study concentrated on stages I and II patients, where relapse rates are lower, this focus inherently led to a smaller sample size. Despite this, the findings provide valuable insights into the potential role of DNA methylation markers for monitoring disease progression and guiding personalized treatment in Wilms tumor patients.

**Graphical abstract:**

Genome methylation analysis of WT tumor and normal tissues from complete remission and relapse patients revealed 14 differentially methylated probes (DMPs) and three differentially methylated regions (DMRs) in tumor samples between both groups. Most DMPs demonstrated strong predictive performance for overall and event-free survival. RNA expression analysis showed elevated *INS-IGF2* levels in relapse tumor tissue, highlighting its role in WT progression.
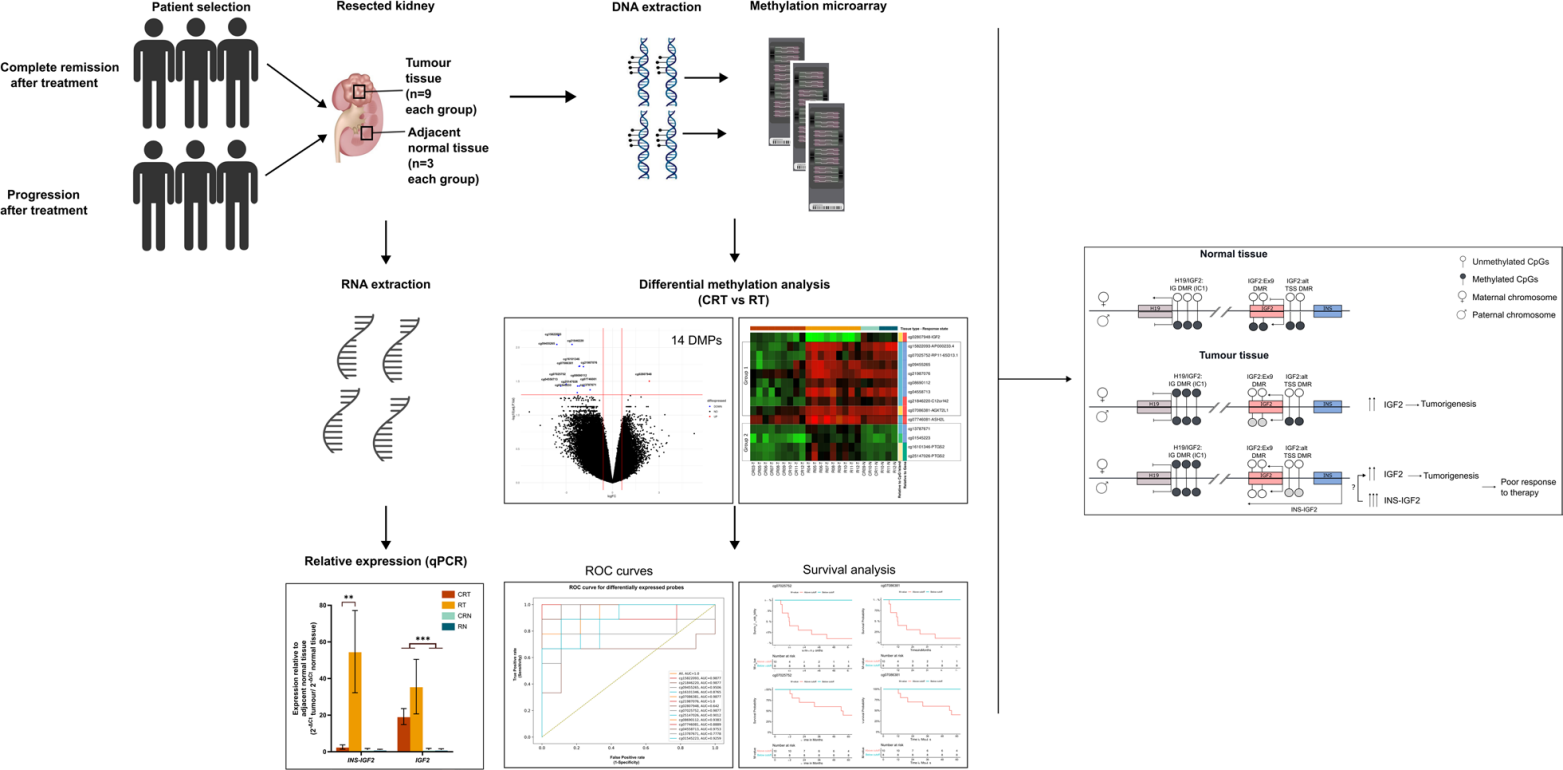

**Supplementary Information:**

The online version contains supplementary material available at 10.1186/s13148-024-01775-y.

## Background

Wilms tumor (WT), also known as nephroblastoma, is the most commonly observed renal tumor within the genitourinary tract of children and constitutes 5% of all childhood malignancies [1]. WTs are usually classified by location, spread and number of kidneys involved to stages I–V, and by histological subtype to favorable (FHWT) or unfavorable histology (UFHWT) [2]. As per the Children’s Oncology Group (COG) protocol, fully resectable stages I, II and III WT are treated with upfront nephrectomy followed by chemo-radiotherapy. For inoperable stage III, as well as stages IV, and V, preoperative chemotherapy is required to shrink the tumor before surgery. Despite high overall survival (OS) rates of 80–85% [3, 4], even reaching 90% in high-income countries, approximately 15% of treated patients experience relapse. Recurrence of the disease significantly lowers overall survival to around 50% and complicates patient outcomes [5].

Similar to other cancers, requiring multiple hits, several genetic and epigenetic changes were identified to be involved in WT etiology [6]. Underlying predisposition syndromes and genetic changes were identified in up to 15% of the WT cases, serving as the first hit for cancer development. One of the first genes identified to predispose to WT development is the Wilms tumor 1 gene, *WT1*, a transcriptional factor crucial for normal kidney development [7]. Developmental syndromes such as WAGR (Wilms’ tumor, aniridia, genital anomalies and retardation) and DDS (Denys–Drash syndrome) are associated with *WT1* pathogenic variations and were shown to have an increased risk of WT development (30–75%) and lower mean ages of WT diagnosis [8, 9]. Another major predisposition factor for WT development is the loss of imprinting (LOI) at imprinting control regions 1 and 2, (IC1 and IC2) on chromosome 11, which causes an activated expression of *IGF2*, a cellular proliferation factor, and reduced expression of H19, a long noncoding RNA that regulates cellular growth [10]. Syndromes with LOI abnormalities, such as BWS (Beckwith–Wiedemann syndrome) subtype characterized by IC1 gain of methylation, have an increased risk for WT (~ 25%) [10, 11]. Other predisposition factors include *CTR9* mutations, DICER1 syndrome and *TRIM28* mutation, which mostly affect chromatin or methylation levels at the chromatin level [12].

One of the earliest identified epigenetic hallmarks of cancer is the hypomethylation of repetitive regions across the entire genome. Paradoxically, it was later identified to be associated with regions of hypermethylation in promoters of tumor suppressor genes, leading to their silencing. Aberrant DNA methylation is an early event in tumorigenesis, occurring not only in cancerous tissues but also in abnormal non-neoplastic tissues [13, 14]. With a focus on focal hypermethylation of tumor suppressor gene promoters, DNA demethylating agents have been approved as cancer chemotherapeutic agents either alone or in combination, in the treatment of myelodysplastic syndromes (MDS), acute myeloid leukemia and chronic myelogenous leukemia [15, 16]. This, however, has to be approached with caution, because induced DNA hypomethylation has been proven to cause carcinogenesis or tumor progression in many models [17, 18].

The early onset of DNA methylation changes make detection of these an attractive diagnostic and prognostic biomarker. Several studies link DNA methylation changes with patient survival and prognosis in several cancers such as brain, breast, lung and head and neck cancers [19–23]. Because of the strong association of aberrant DNA methylation and WT development, several studies investigated its association with WT progression, prognosis and patient survival. Focal methylation changes, such as reduced *P73* promoter methylation, was found to be associated with poor prognosis [24]. Other studies have linked overall methylation patterns with risk of disease progression and have formed stratification criteria based on methylation patterns [25, 26]. These studies have helped define specific methylation markers to identify high-risk groups, but still little is known about the causes of progression in a subset of the otherwise considered low-risk WTs.

In this study, we aimed to identify biomarkers for relapse in patients with stages I and II FHWT. We used microarray technology to explore methylation patterns in both WT and adjacent healthy tissues from excised kidneys prior to any chemotherapy treatment. We report several differentially methylated regions (DMRs) that show potential as prognostic biomarkers for disease relapse and overall patient survival. Finally, to explain the mechanistic role of DNA methylation changes in WT relapse, we explore the genes controlled by the differentially methylated *IGF2* region and show elevated expression of *INS-IGF2* transcript in tumor tissue of patients who have relapsed after treatment.

## Methods

### Ethical approval and sample collection

Informed consents, approved by the Institutional Research Ethics Board (IRB) at CCHE 57357, were collected from guardians of patients with stage I or II WT with favorable histology. Tumor and normal FFPE tissue samples were collected from the Pathology Department from two cohorts of patients: nine who achieved complete remission (CR) and were followed up for at least 6 years post-treatment (mean follow-up period: 93 months), and nine who experienced recurrent disease within 5 years of treatment (mean relapse time: 16 months). The clinicopathological characteristics of the patients recruited are listed in Table 1.

**Table 1:** Clinicopathological characteristics of enrolled WT patients

	Complete remission group (n = 9)	Relapse group (n = 9)	Complete remission normal samples (n = 3)	Relapse group normal samples (n = 3)
Age (years) (Mean ± SD)	2.75 ± 1.68	4.07 ± 3.17	2.99 ± 1.72	6.47 ± 4.4
Gender (Female, Male)	6, 3	3, 6	2, 1	0, 3
Stage (I, II)	4, 5	5, 4	1, 2	2, 1
5-year overall survival (% survival)	100%	33%	100%	0%

### Sample preparation

Ten sections of 8 μm thickness were obtained from formalin-fixed, paraffin-embedded (FFPE) tissue blocks of the initially resected kidney samples (prior to any chemotherapy), from eighteen tumor tissues (nine from complete remission patients and nine from relapse patients) and six adjacent normal tissues (three from complete remission patients and three from relapse patients). Genomic DNA was extracted from the FFPE sections using QIAamp DNA FFPE tissue kit (Qiagen) according to the manufacturer’s instructions. DNA was quantified using the Denovix Fluorometer (dsDNA High Sensitivity). The quality of the extracted FFPE DNA samples was assessed by Illumina FFPE QC kit (Illumina Inc.). Bisulfite conversion of extracted DNA was performed using the EZ DNA methylation kit (D5002, Zymo Research) according to the manufacturer’s instructions using the alternative incubation conditions recommended for the Illumina Infinium methylation arrays. Bisulfite-converted FFPE DNA was then restored with Infinium HD FFPE DNA Restore Kit (WG-321-1002, Illumina Inc.). Restored bisulfite-modified DNA samples were hybridized to the Illumina Infinium Human Methylation EPIC bead chips and scanned using the Illumina iScan microarray scanner (Illumina Inc.) according to the manufacturer’s recommendations.

### Bioinformatics analysis

**Fig. 1:**
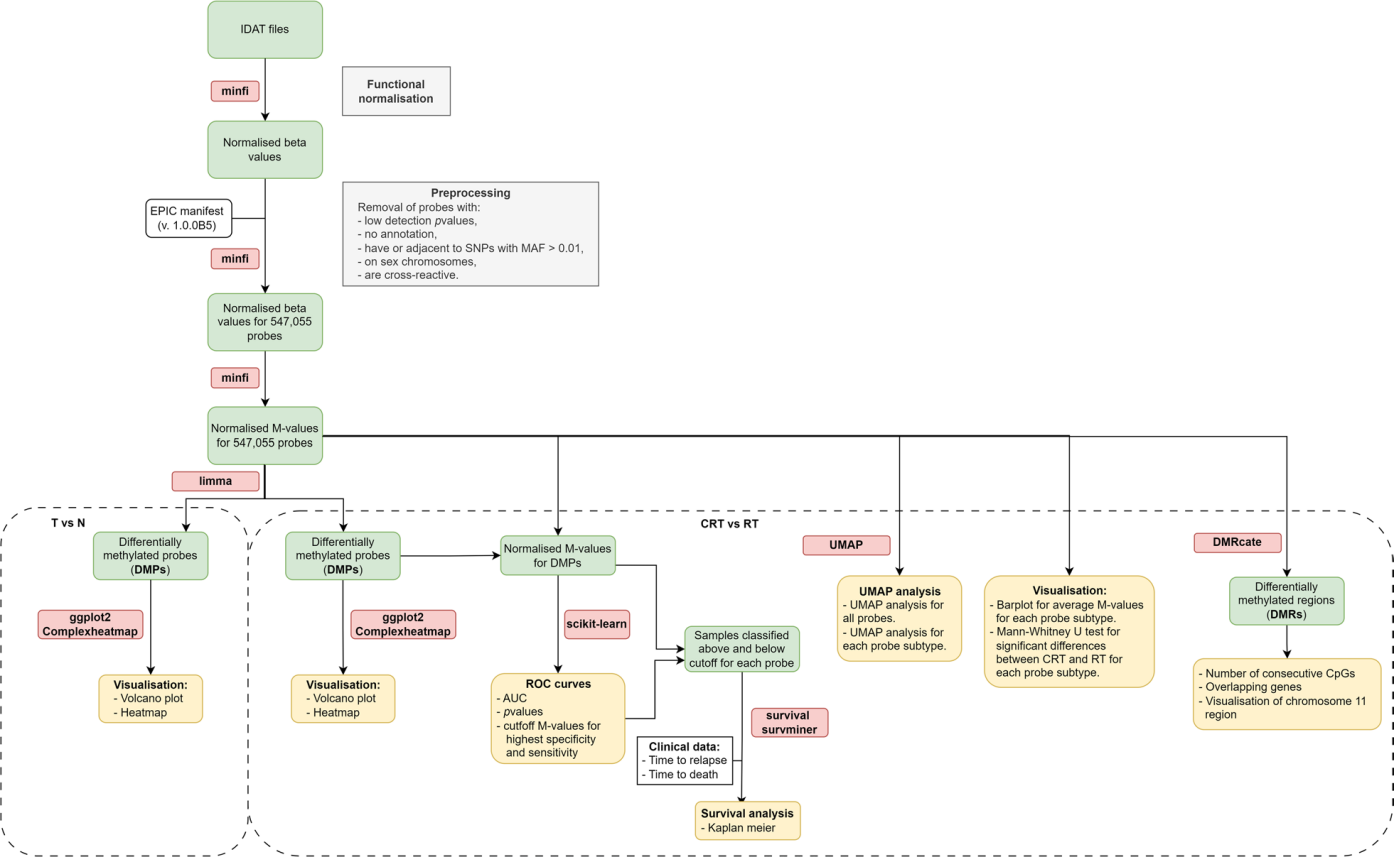
Methylation analysis bioinformatics workflow. The workflow includes data processing milestones (normalization, preprocessing, M-value calculations, followed by differential expression, ROC curves and survival analysis). The diagram was created using draw.io software

The bioinformatics workflow is summarized in Fig. 1. R software (v4.3.1) and minfi R package (v1.44.0) [27] were used to load the Illumina Human Methylation EPIC intensity data files (IDAT) and check their quality. To overcome technical variations between probe types I and II, functional normalization [28] was employed. Probes with detection *p* values less than 0.01 were removed. EPIC Manifest (v. 1.0.0B5) was used for probe annotation, with the exclusion of probes with polymorphism in their CG sites with minor allele frequency (MAF) larger than 0.01 (i.e., non-rare polymorphism), probes present on single-nucleotide polymorphism sites (SNPs) [29] or sex chromosomes and probes matching more than one genomic site (i.e., cross-reactive probes) [30, 31]. Only preprocessed probes with annotation data were selected, and 547,055 probes were ultimately chosen for further analysis.

M-values were calculated, and differential analysis was performed using the limma R package (v3.54.2). Our comparisons included relapsed tumor samples (RT) versus complete remission tumor samples (CRT), as well as tumor (RT and CRT) versus normal tissue samples (CRN and RN). Differentially methylated probes (DMPs) from both comparisons were determined based on an absolute log2 fold change (abs(log2FC) ≥ 0.5), with a false discovery rate (FDR) of < 0.05. Volcano plots were generated from the normalized M-values for DMPs, and a heatmap was plotted for visualization.

Further investigations were performed to compare CRT and RT samples. To identify overall differences, UMAP analysis was performed using UMAP R library (v0.2.10.0.) with number of neighbors = 8, and using the first two components. To test differences for each probe subtype, average M-value for each probe subtype was calculated for each sample group, statistical significance was analyzed using Mann–Whitney U-test, and was considered significant if *p* value < 0.05. To probe the utility of the identified DMPs as biomarkers, logistic regression was performed using the Scikit-learn (v1.3.2) library in Python, and the resulting model was used to plot receiver operating characteristic (ROC) curves. ROC curves were also plotted using IBM SPSS Statistics v22.0 to identify M-value cut-offs showing the highest sensitivity and specificity for each probe. Survival analysis was conducted using the survival (v3.5.7) and survminer (v0.4.9) R packages, based on the cut-offs for each probe obtained earlier. To test the relationship between methylation levels (M-values) in each of the fourteen DMPs and time to relapse, linear regression was performed, with time to last follow-up used in complete remission patients. To further investigate DMRs, we utilized the dmrcate function from the DMRcate [32] package (v2.12.0), using a lambda of 1000 and C of 2, while consecutive probes were set to true.

### RNA expression

Further sections were obtained from the same FFPE samples as previous. RNA was extracted from the FFPE sections using AmoyDx FFPE RNA extraction kit (AmoyDx, Xiamen, China) according to manufacturer’s instructions. RNA was quantified using the Qubit Fluorometer (RNA broad range). The quality of the RNA was assessed using Agilent RNA 6000 Nano (Agilent technologies, USA). cDNA was generated using High-Capacity cDNA Reverse Transcription Kit (Thermo Fisher Scientific, USA). RT-PCR was used to analyze relative expression levels using Maxima SYBR Green qPCR Master Mix (Thermo Fisher Scientific, USA). Ct values were analyzed relative to GAPDH Ct values. Primers used for GAPDH are FP: TGCCCTCAACGACCACTTTG and RP: CCACCACCCTGTTGCTGTAG, for IGF2; FP: TGGCATCGTTGAGGAGTGCTGT and RP: ACGGGGTATCTGGGGAAGTTGT, and for INS-IGF2; FP: ATCATCGTCCAGGCAGTTTCGG and RP: ACACAAGCTCGGTGGTGACTCT. ΔCt was calculated as Ct (IGF2 or INS-IGF2) − Ct (GAPDH) and then converted to 2^−ΔCt^. Expression is reported relative to adjacent normal tissue for each group separately; CRT relative to CRN and RT relative to RN. Statistical significance was analyzed using Mann–Whitney U-test and was considered significant if *p* value < 0.05.

## Results

### Global hypomethylation and focal hypermethylation are hallmarks of WT pathogenesis

**Fig. 2:**
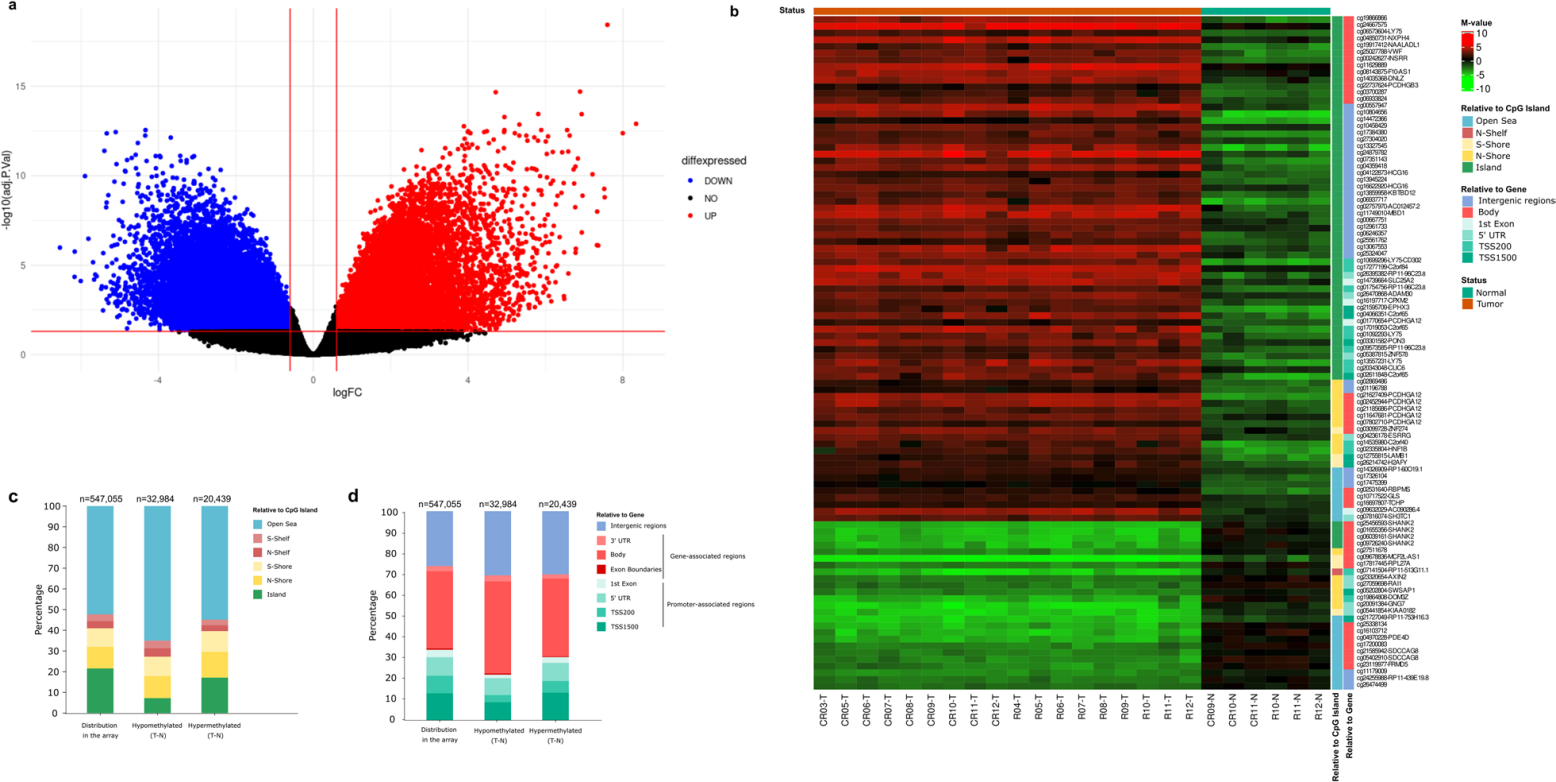
Global differences in methylation levels between WT and adjacent normal tissues. **a** Volcano plot of methylation differences between WT and adjacent normal tissues, showing a total of 53,423 DMPs, 32,984 hypomethylated and 20,439 hypermethylated. **b** Heatmap showing the top 100 DMPs, annotated for relation to CpG island, and genes. **c**–**d** Stacked bar plot showing localization of hyper- and hypo-methylated CpG sites in WT tissue relative to CpG islands (**c**) and to their adjacent genes (**d**)

To observe the overall methylation changes that occur during tumorigenesis in WT, we performed methylation analysis to compare the 18 tumor tissue samples and the six adjacent normal tissue. As previously shown for WT and other cancers, there was a significant difference in methylation levels between normal and cancerous tissues (Fig. 2a). A total of 53,423 probes were significantly differentially methylated between tumor and adjacent normal tissues, and the top 100 are shown in Fig. 2b. The majority of the hypomethylated probes were far from CpG sites, in the open sea regions, and within gene bodies (Fig. 2c and d). Hypermethylated probes, however, were more abundant at promoter-associated regions (Fig. 2c and d).

### Methylation differences can predict WT relapse

**Fig. 3:**
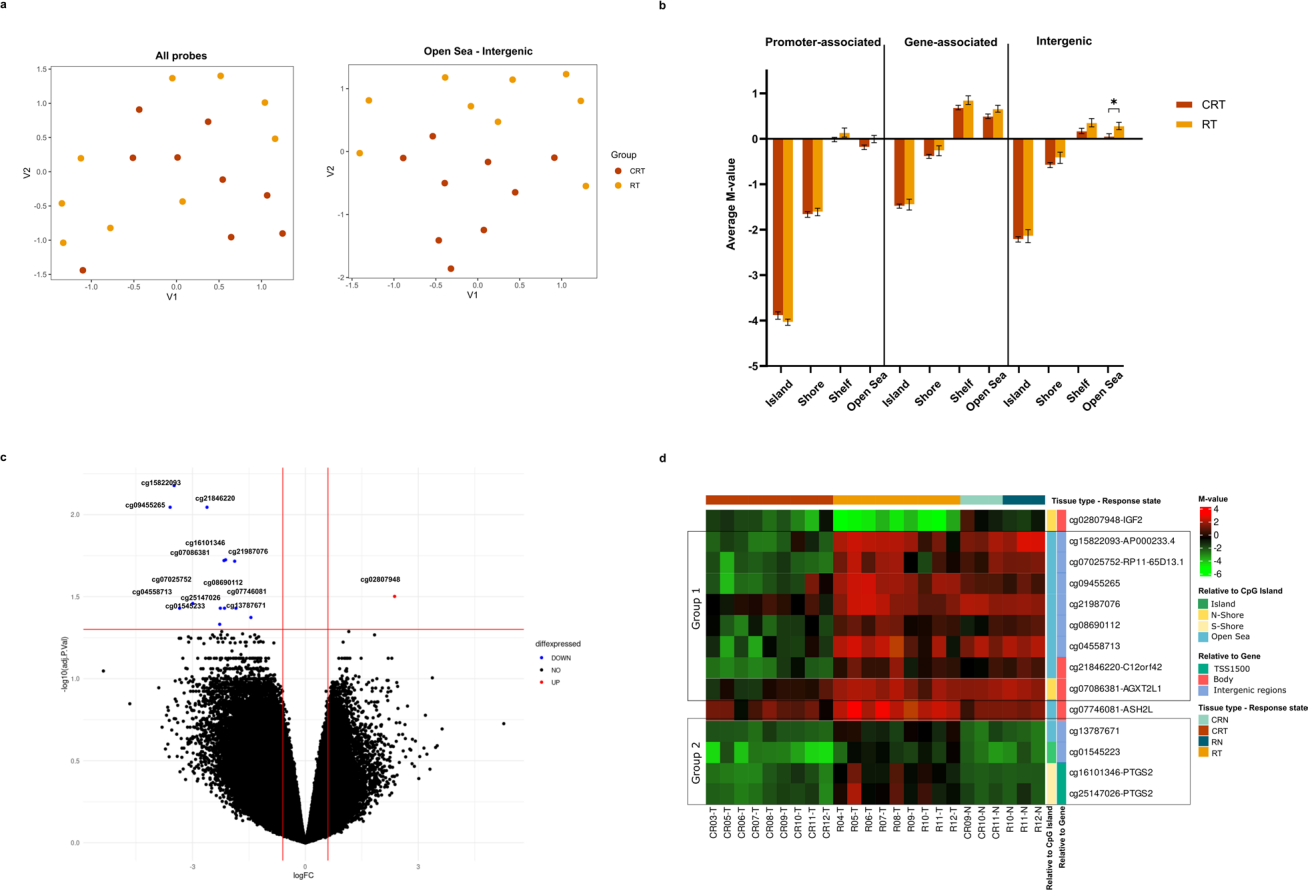
Methylation differences between relapse and complete remission patients. **a** UMAP clustering of methylation probes for CRT and RT samples for all probes, and for probes in the open sea intergenic regions. **b** Bar plot of average M-values for each probe subtype and sample group, statistical significance is tested using Mann–Whitney U-test, one asterisk for *p* value < 0.05. **c** Volcano plot of methylation differences between CR and R patients showing 14 DMPs; 13 hypomethylated and 1 hypermethylated. **d** Heatmap showing M-values across different samples in all four groups, annotated for relation to genes and to CpG islands

In an attempt to discover methylation differences that can help predict relapse in stages I and II FHWT patients, we compared tumor DNA methylation levels on upfront nephrectomy samples between nine patients who remained in complete remission for at least 5 years post-treatment, and nine who had relapsed within 5 years. When analyzing all probes together, we could not see distinct clustering between relapse tumor samples (RT) and complete remission tumor samples (CRT) using UMAP clustering (Fig. 3a). When the probes were separated based on their location relative to genes and CpG islands, we observed clustering between RT and CRT samples only in the open sea intergenic regions (Fig. 3a and Supplementary Fig. 1), where CRT samples showed significantly lower methylation levels (Fig. 3b, Mann–Whitney U-test, *p* value < 0.05).

Fourteen DMPs between relapse (R) and complete remission (CR) patients were identified (Fig. 3c and d), with almost all of them hypermethylated in relapse patients. When analyzed in the context of adjacent normal tissue, these DMPs can be divided into two major groups. The first group of probes shows pronounced hypomethylation in complete remission patients, where the majority of which are in open sea, intergenic and gene body regions. This includes probes in the gene body of *C12orf42* and *AGXT2L1*, and in the proximity of *RP11-65D13.1* and the lncRNA AP000233.4. The second group of probes shows hypermethylation in relapse patients. This includes probes in the promoter-associated region of the inflammatory gene prostaglandin endoperoxide synthase 2, *PTGS2*. A notable probe is cg02807948, which is the only probe that is significantly hypomethylated in relapse patients, and it resides within the imprinted IGF2:Ex9-DMR (DMR2).

### Specific methylation foci represent promising biomarkers of the disease prognosis

**Fig. 4:**
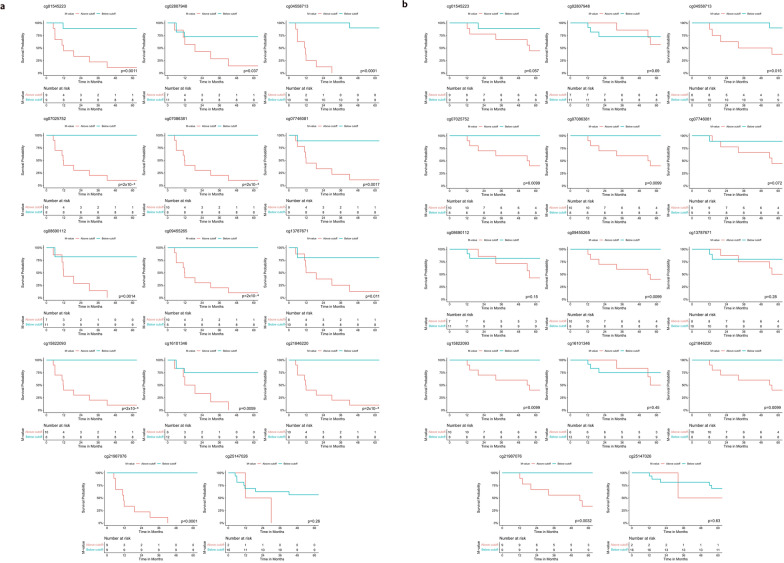
DMPs can be used as prognostic biomarkers for predicting event-free (EFS) and overall survival (OS). Kaplan–Meier survival analysis was performed after dividing samples based on cut-offs obtained from ROC curves. **a** Event-free survival differences are plotted for the 14 probes, with *p* values calculated using the log-rank test. **b** Overall survival differences are plotted for the 14 probes with significant *p* values (< 0.05) from the log-rank test

The potential of utilizing these probes as biomarkers for disease prognosis was explored through logistic regression models, followed by the generation of receiving operator characteristic (ROC) curves. The area under curve (AUC) with *p* values and cut-off M-values showing the best sensitivity and specificity are reported in Table 2. Most of the probes showed AUC > 0.8 and *p* value < 0.05, with the best performing probe, cg21987076, showing an AUC = 1. Forming a panel with all 14 probes as a prognostic marker showed AUC of 1. By the cut-off value that was determined by the ROC curve, patients were divided into two groups, those with M-values above and below the cut-off. Kaplan–Meier survival analysis and log-rank test were used to compare the difference in event-free survival (EFS) and overall survival (OS) times between both groups. Probes with their log-rank test *p* values < 0.05 for both EFS and OS are shown in Fig. 4. Once again, the best performing probe is cg21987076, showing the greatest difference in EFS and OS rates between both groups. Altogether, for all probes except cg02807948, a higher M-value above the cut-off is associated with poor prognosis.

**Table 2:** Metrics from logistic regression and ROC curve analysis

Probe ID	AUC	*p* value	Cut-off (M-value)	Sensitivity (%)	Specificity (%)
cg21846220	0.988	4.87 × 10^–4^	> − 1.276	100	88.9
cg15822093	0.988	4.87 × 10^–4^	> − 0.873	100	88.9
cg09455265	0.951	1.27 × 10^–3^	> 0.159	100	88.9
cg16101346	0.877	7.08 × 10^–3^	> − 1.26	66.7	100
cg07086381	0.988	4.87 × 10^–4^	> 0.367	100	88.9
cg21987076	1.000	3.49 × 10^–4^	> 0.705	100	100
cg02807948	0.642	0.31	< − 3.27	64.2	31.0
cg07025752	0.988	4.87 × 10^–4^	> − 1.913	100	88.9
cg25147026	0.901	4.11 × 10^–3^	> 0.159	100	88.9
cg08690112	0.938	1.72 × 10^–3^	> − 0.399	77.8	100
cg07746081	0.889	5.41 × 10^–3^	> 1.326	88.9	88.9
cg04558713	0.975	6.75 × 10^–4^	> − 0.164	88.9	100
cg13787671	0.778	0.047	> − 1.924	77.8	88.9
cg01545223	0.926	2.32 × 10^–3^	> − 2.694	88.9	88.9

Area under the ROC curve (AUC) for all fourteen methylation probes, the cut-off M-values and their associated sensitivity and specificity

To identify if the level of methylation affects the time for disease relapse, we performed linear regression analysis for the fourteen DMPs. We identified three probes that correlate with time to disease relapse, cg15822093 (R^2^ = 0.83, *p* value = 1.6 × 10^–7^), cg07086381 (R^2^ = 0.765, *p* value = 2.03 × 10^–6^) and cg21987076 (R^2^ = 0.717, *p* value = 9.23 × 10^–6^). Altogether, these findings suggest that these probes, especially cg21987076, hold significant promise as prognostic biomarkers, with potential clinical implications for predicting survival outcomes and disease relapse.

### Hypomethylation in IGF2:Ex9-DMR causes elevated expression of INS-IGF2 contributing to resistance to chemotherapy

We investigated the methylation differences across consecutive probes, defining regions of differential methylation (DMRs). When comparing CRT vs RT samples; we identified three significant DMRs (Table 3) on chromosomes 13, 11 and 1. These DMRs overlap with several genes, including ncRNAs genes; small nucleolar RNA (*SNORD38*), long non-coding RNA (*LINC0559*) and microRNA (*MIR483*); pseudogenes *PEX12P1*, *FAR1P1*, *KRT8P27* and *RNA5SP34* and proliferatorive genes such as insulin growth factor 2 (*IGF2*) and BMP/Retinoic Acid Inducible Neural Specific 3 (*BRINP3*). To further characterize methylation changes relative to surrounding normal tissue, we compared RT vs RN samples and CRT vs CRN samples (Supplementary Table 2). The RT vs RN comparison yielded 1543 DMRs, while CRT vs CRN yielded 296 DMRs, suggesting more extensive methylation changes in relapse samples.

**Table 3:** Differentially methylated regions and overlapping genes between CR and R patients

DMR	Chromosome	Start position	End position	Width	Number of CpGs	Min. smoothed FDR	Overlapping genes
1	chr13	90,488,675	90,883,434	394,760	8	1.54 × 10^–44^	*SNORD38*, *PEX12P1, FAR1P1, KRT18P27, LINC00559, RNA5SP34*
2	chr11	2,154,432	2,156,282	1851	4	1.31 × 10^–40^	*IGF2, INS-IGF2, MIR483*
3	chr1	190,232,637	190,323,079	90,443	3	5.36 × 10^–40^	*RP11-547I7.1, BRINP3*

**Fig. 5:**
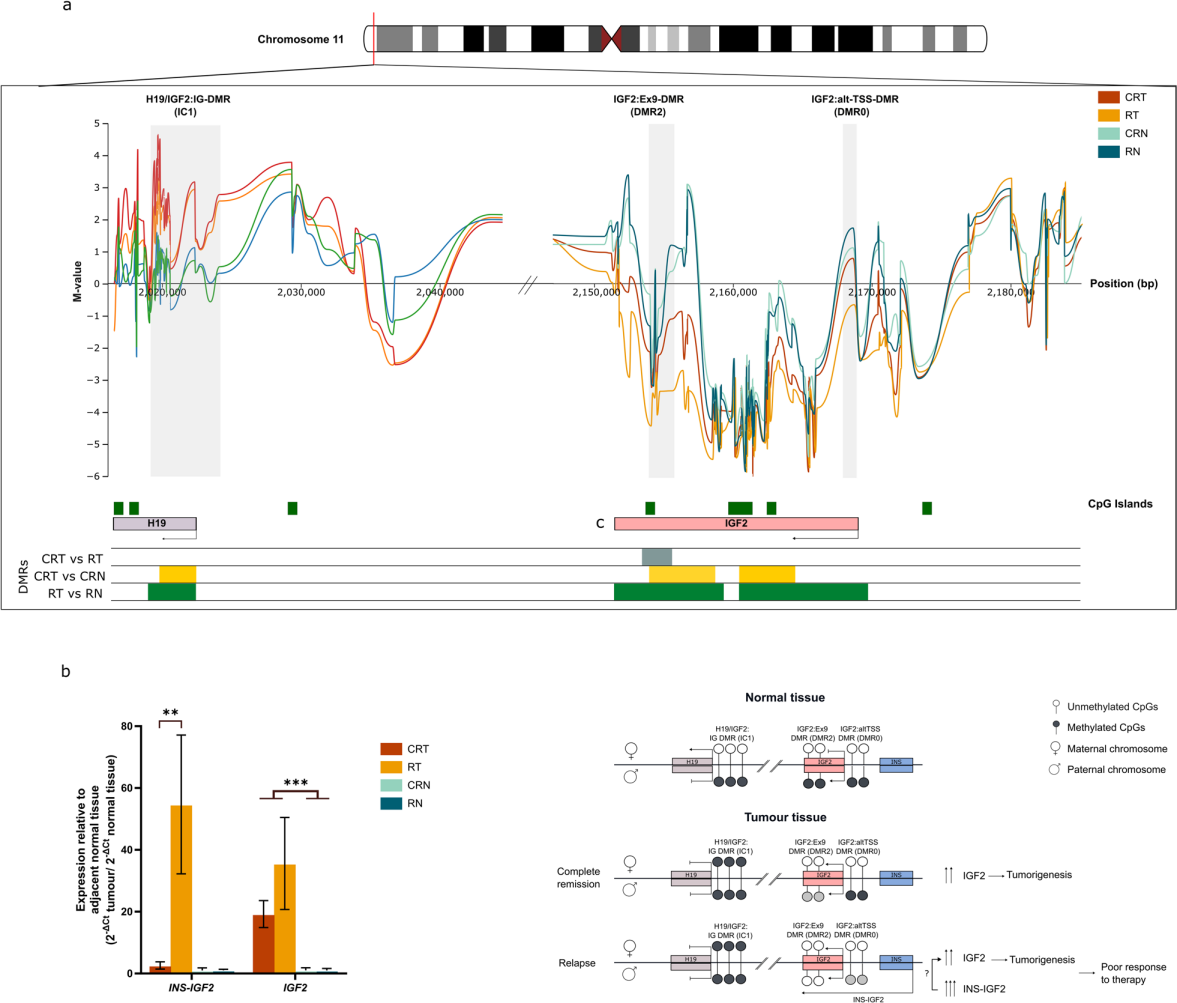
Loss of methylation in IGF2:Ex9-DMR causes elevated expression of INS-IGF2 contributing to resistance to chemotherapy **a** Visualization of methylation levels and differentially methylated regions in each of the four subgroups across three imprinting-associated DMRs on chromosome 11. Loss of methylation is observed in tumor samples at IC1 and DMR2, whereas DMR0 shows loss of methylation in tumor samples of relapse patients only. Coordinates for each DMR (GRCh37) are as follows: H19/IGF2 (IC1): chr11:2018812-2024740; IGF2:Ex9-DMR (DMR2): chr11:2153991-2155112 and IGF2:alt-TSS-DMR (DMR0): chr11:2168333-2169768. **b** RNA expression levels of *IGF2* and *INS-IGF2* by real-time PCR. ΔCt values are calculated as (mean Ct _*IGF2*/*INS*-*IGF2*_ − Ct _*GAPDH*_) and converted to 2^−ΔCt^. For tumor tissue from each group, relapse and complete remission patients, 2^−ΔCt^ values reported relative to normal adjacent tissue. Data are presented as mean ± standard error of mean (SEM) relative expression: **p < 0.01; ***p < 0.005 (by Mann–Whitney U-test). **c** Illustrative model describing how loss of methylation in IGF2:Ex9-DMR leads to poor response to therapy by increasing INS-IGF2 expression levels

Due to its established association with WT, we examined chromosome 11 in more detail. Figure 5a shows the M-values across this region for all three comparisons, highlighting three known imprinting-associated DMRs (iDMRs); H19/IGF2:IG-DMR (ICR1) and IGF2:Ex9-DMR (DMR2) and IGF2:alt-TSS-DMR (DMR0). Individual sample M-values for each group across this region are presented in Supplementary Table 1. As shown in Table 3, Supplementary Table 2 and Fig. 5a, both RT and CRT exhibit gain of methylation in H19/IGF2:IG-DMR (IC1), whereas the other two secondary iDMRs, IGF2:Ex9-DMR (DMR2) and IGF2:alt-TSS-DMR (DMR0), show variable changes. Specifically, IGF2:Ex9-DMR (DMR2) showed loss of methylation in both CRT and RT as compared to their respective normal tissues with a greater effect in RT samples (Supplementary Table 2, Fig. 5a). IGF2:alt-TSS-DMR (DMR0) exhibits loss of methylation only in RT samples (Supplementary Table 2 and Fig. 5a). To assess the functional impact of these methylation changes on RNA expression, we examined *IGF2* and *INS*-*IGF2* expression via real-time PCR. Ct values of *IGF2* and *INS-IGF2* normalized to GAPDH, were used to calculate ΔCt values, and relative expression was derived by dividing 2^−ΔCt^ for tumor vs adjacent normal tissue within each group. Our results show a 20-fold increase in *INS-IGF2* expression levels in tumor tissues of relapse patients than that of complete remission patients (*p* value < 0.001), whereas *IGF2* expression differs by less than twofold between these groups (Fig. 5b). Altogether, this indicates that while there is a similar gain of methylation at IC1, the loss of methylation in DMR2 and DMR0 correlates with higher *INS-IGF2* levels, potentially contributing to chemotherapy resistance and tumor relapse (Fig. 5c).

## Discussion

Although current WT therapies achieve survival rates of over 85%, long-term survivors of WT are at increased risk of treatment-related morbidity and mortality [1, 33]. Treatment complications include cardiotoxicity from anthracycline treatment, musculoskeletal effects and the development of secondary malignant neoplasms [34]. Survival rates also significantly decrease to around 30% in cases of tumor relapse [1, 2, 35]. Current efforts focus on the early detection of prognostic markers to effectively adjust treatment approaches, minimize treatment doses for low-risk patients and enable early identification of relapse potential to optimize treatment strategies.

In our study, we used methylation microarrays to explore global methylation levels in both tumor tissues and adjacent normal tissues from initially resected kidneys of stages I and II FHWT patients. We report both regions of hypomethylation and regions of focal hypermethylation in tumor tissue compared to adjacent normal tissues. The majority of the hypomethylation is in open sea regions, as well as in intergenic and gene body associated regions, whereas hypermethylation was mostly observed in CpG islands and promoter-associated regions. This is consistent with other studies that report hypomethylation as a hallmark of cancer, particularly in intergenic and open sea regions, leading to the activation of gene expression from repeat regions [36], as well as focal hypermethylation [37], inhibiting the expression of tumor suppressor genes.

The potential of DNA methylation as a prognostic marker has been investigated in numerous studies, particularly due to the early onset of these changes. Research on WT has examined methylation markers across various WT samples [24–26] and showed that most methylation differences are influenced by histopathological subtype and tumor stage. In this study, we specifically focus on low-risk stages I and II WT patients, to identify the earliest methylation biomarkers for relapse. Interestingly, we observe a higher hypomethylation pattern particularly in intergenic open sea regions, as a good prognostic indicator for complete remission following kidney resection and chemotherapy. This is in contrast with several studies that report the association of hypomethylation with more progressive disease [36, 38, 39]. Furthermore, we demonstrate the effectiveness of fourteen identified DMPs as discriminative biomarkers. By using cut-offs from ROC curves, we stratified the samples and found correlations with overall survival and event-free survival. Moreover, our analysis indicates that methylation levels may not only predict the occurrence of disease relapse but also the timing of relapse. Through linear regression, we identified three probes—cg15822093, cg07086381 and cg21987076—that significantly correlate with time to relapse, highlighting their potential as prognostic markers for disease progression. Several studies have shown that DNA methylation can be detected in cell-free circulating DNA (cfDNA) [40–42], highlighting its potential as a valuable biomarker in various cancers, while minimizing the need for invasive tissue biopsies. This suggests that the biomarkers identified in this study could be further evaluated as non-invasive blood-based indicators, enabling real-time monitoring of tumor dynamics and treatment response.

Several of the DMPs identified were located around genes that have reported involvement in carcinogenesis and overall survival. Higher expression of *RP11*-*65D13*.1 is observed in squamous cell carcinoma [43], whereas lower expression of *AGXT2L1* is observed in digestive cancers [44]. Higher expression of the long non-coding RNA AP000233.4, *C12orf42* and *PTGS2* are associated with better survival [45–47]. However, since methylation changes across one or two isolated probes may not reflect on overall gene expression levels, we focused our attention on the DMR involving *IGF2* gene.

The methylation status of the chromosome 11 region, particularly the IC1 located between the *IGF2* and *H19* genes, has been extensively studied. In addition to IC1, two other iDMRs are present at the *IGF2* locus, *IGF2:alt-TSS* (DMR0) and *IGF2:Ex9-DMR* (DMR2). IC1 is imprinted under normal conditions, ensuring the mono-allelic expression of *H19* and *IGF2* with DMR2 also participating in this regulation. Specifically, DMR2 is believed to facilitate paternal expression of *IGF2*, through interacting in its methylated paternal form in *cis* with paternal methylated IC1 activating the expression of *IGF2*, by placing its promoters in close vicinity to the enhancers downstream of *H19* [48]. Loss of imprinting during embryogenesis is observed in syndromic disorders such as BWS and Silver–Russell syndrome (SRS), resulting in over-growth or under-growth disorders, respectively. Loss of imprinting at IC1 is also seen in several cancers such as hepatocellular carcinoma and nephroblastoma, underlying the high prevalence of WT in BWS patients. While IC1 shows gain of methylation in WT of both syndromic and nonsyndromic origins, the status of DMR0 and DMR2 follows a different pattern. DMR0 was shown to be hypomethylated in WT patients, but hypermethylated in BWS patients [49], whereas DMR2 shows gain of methylation in BWS patients with maternal origin gain of methylation at IC1 [50].

Here, we report, as expected, the gain of methylation at the IC1 region in tumor samples from both groups compared to the adjacent normal tissues. In tumor tissues of relapse patients, we also observed loss of methylation of DMR2, which was significantly greater than that seen in complete remission patients, in addition to a loss of methylation at DMR0, which was not observed in complete remission patients. These findings suggest a new role for DMR0 and DMR2 in chemoresistance in WT patients. We investigated the possible transcription effects of these methylation changes, on *IGF2* and the typically unexpressed *INS*-*IGF2* fusion transcript. We observed a 20-fold higher expression level of *INS-IGF2* transcript in tumor tissues of relapse patients when compared to complete remission patients, in contrast with a modest twofold higher expression level of *IGF2*. The overexpression of *INS*-*IGF2* has been reported in cancers such as pheochromocytomas [51] and was shown to promote cellular proliferation and migration in lung cancer [52]. The proposed mechanism of action for the *INS-IGF2* transcript in promoting cancer progression involves its action as a *cis*-acting regulation of the *IGF2* gene, resulting in its overexpression. However, our findings indicating only modest changes in *IGF2* expression suggest that *INS-IGF2* might exert its proliferative effects through alternative, unexplored mechanisms. The loss of methylation at DMR0 and DMR2 is a likely reason for the increased expression of the *INS-IGF2* fusion transcript. DMR0 is located between the *INS* and *IGF2* genes, and its demethylation could potentially lead to run-off transcription, producing the fusion transcript. Loss of methylation at DMR2, while IC1 remains methylated—unlike the typical pattern in paternal IGF2 expression—may induce abnormal chromatin changes that promote the expression of the *INS-IGF2* fusion. Additionally, DMR2 overlaps enhancer regions, and several studies report conflicting theories on the relationship between DNA methylation and enhancer activity or the recognition of enhancers as promoter elements [53–55].

Naturally, this study has inherent limitations that must be acknowledged. Due to the focus on identifying early biomarkers of WT disease progression, the study concentrated primarily on stages I and II FHWT patients. This choice limited the sample size, given the rarity of WT and the low relapse rates among these stages. Additionally, the observed methylation differences may be specific to Egyptian pediatric patients. We also noted significant discrepancies in age and gender distribution between the complete remission and relapse groups, which may impact the interpretation of our findings. Future validation studies, requiring multi-center collaboration, are essential to obtain a larger sample size, address potential confounding factors such as age and gender and confirm the utility of DNA methylation as a prognostic marker. Altogether, despite the small sample size and these limitations, this study provides valuable preliminary insights and lays the groundwork for future research with larger cohorts.

## Conclusions

The analysis of DNA methylation patterns and *INS-IGF2* expression in early-stage Wilms tumor patients with favorable histology reveals significant potential for these biomarkers in predicting relapse and disease progression. The identification of methylation changes at the chromosome 11 DMR2 and the association with increased *INS-IGF2* expression provides mechanistic insights into the molecular mechanisms underlying disease progression. Further validation and exploration of these biomarkers could lead to more targeted and personalized treatment strategies, ultimately improving long-term outcomes for Wilms tumor patients.

## Supplementary Information

Additional file 1.

Additional file 2.

Additional file 3.

## Data Availability

All data generated during this study are included in this article and its supplementary files. Raw methylation data were deposited at the Gene Expression Omnibus (GEO) under accession number GSE269241.

## Abbreviations

WT: Wilms tumor
DMP: Differentially methylated probe
COG: Children’s Oncology Group
OS: Overall survival
WAGR: Wilms’ tumor, aniridia, genital anomalies and retardation
DDS: Denys–Drash syndrome
LOI: Loss of imprinting
IC1, IC2: Imprinting control regions 1 and 2
BWS: Beckwith–Wiedemann syndrome
MDS: Myelodysplastic syndromes
IRB: Institutional Research Ethics Board
IDAT: Intensity data files
MAF: Minor allele frequency
SNPs: Single-nucleotide polymorphism sites
RT: Relapsed patients-tumor tissue
CRT: Complete remission patients-tumor tissue
RN: Relapse patients-normal tissue
CRN: Complete remission patients-normal tissue
FDR: False discovery rate
ROC: Receiver operating characteristic
DMR: Differentially methylated regions
Ct: Cycle threshold
AUC: Area under curve
EFS: Event-free survival
iDMRs: Imprinting-associated DMRs
SEM: Standard error of mean
SRS: Silver–Russell syndrome

## References

[1] Szychot E, Apps J, Pritchard-Jones K. Wilms’ tumour: biology, diagnosis and treatment. Transl Pediatr. 2014;3(1):124–124. 10.3978/J.ISSN.2224-4336.2014.01.09. 10.3978/j.issn.2224-4336.2014.01.09 PMC4728859 26835318

[2] Davidoff AM. Wilms’ tumor. Curr Opin Pediatr. 2009;21(3):357–64. 10.1097/MOP.0B013E32832B323A. 19417665 10.1097/MOP.0b013e32832b323a PMC2908383

[3] Soliman RM, Elhaddad A, Oke J, et al. Temporal trends in childhood cancer survival in Egypt, 2007 to 2017: a large retrospective study of 14 808 children with cancer from the Children’s Cancer Hospital Egypt. Int J Cancer. 2021;148(7):1562–74. 10.1002/ijc.33321. 32997796 10.1002/ijc.33321

[4] Asfour HY, Khalil SA, Zakaria AS, Ashraf ES, Zekri W. Localized Wilms’ tumor in low-middle-income countries (LMIC): how can we get better? J Egypt Natl Canc Inst. 2020. 10.1186/s43046-020-00043-3. 32794016 10.1186/s43046-020-00043-3 PMC13317114

[5] Groenendijk A, Spreafico F, de Krijger RR, et al. Prognostic factors for Wilms tumor recurrence: a review of the literature. Cancers. 2021;13(13):3142. 10.3390/CANCERS13133142/S1. 34201787 10.3390/cancers13133142 PMC8268923

[6] Sutherl JV, Bailar JC. The multihit model of carcinogenesis: etiologic implications for colon cancer. J Chronic Dis. 1984;37(6):465–80. 10.1016/0021-9681(84)90030-4. 6725500 10.1016/0021-9681(84)90030-4

[7] Frequent Association of β-Catenin and WT1 Mutations in Wilms Tumors1 | Cancer Research | American Association for Cancer Research. Accessed 27 May 2024. https://aacrjournals.org/cancerres/article/60/22/6288/506926/Frequent-Association-of-Catenin-and-WT1-Mutations.

[8] Scott RH, Stiller CA, Walker L, Rahman N. Syndromes and constitutional chromosomal abnormalities associated with Wilms tumour. J Med Genet. 2006;43(9):705–15. 10.1136/JMG.2006.041723. 16690728 10.1136/jmg.2006.041723 PMC2564568

[9] Huff V. Wilms’ tumours: about tumour suppressor genes, an oncogene and a chameleon gene. Nat Rev Cancer. 2011;11(2):111–21. 10.1038/NRC3002. 21248786 10.1038/nrc3002 PMC4332715

[10] Anvar Z, Acurzio B, Roma J, Cerrato F, Verde G. Origins of DNA methylation defects in Wilms tumors. Cancer Lett. 2019;457:119–28. 10.1016/J.CANLET.2019.05.013. 31103718 10.1016/j.canlet.2019.05.013

[11] Brzezinski J, Shuman C, Choufani S, et al. Wilms tumour in Beckwith-Wiedemann Syndrome and loss of methylation at imprinting centre 2: revisiting tumour surveillance guidelines. Eur J Hum Genet. 2017;25(9):1031. 10.1038/EJHG.2017.102. 28699632 10.1038/ejhg.2017.102 PMC5558170

[12] Yang Y, Tan S, Han Y, et al. The role of tripartite motif-containing 28 in cancer progression and its therapeutic potentials. Front Oncol. 2023;13:1–10. 10.3389/fonc.2023.1100134. 10.3389/fonc.2023.1100134 PMC9899900 36756159

[13] Salem C, Liang G, Tsai YC, et al. Progressive increases in de novo methylation of CpG islands in bladder cancer. Cancer Res. 2000;60(9):2473–6. 10811126

[14] Kim YT, Sun JP, Seung HL, et al. Prognostic implication of aberrant promoter hypermethylation of CpG islands in adenocarcinoma of the lung. J Thorac Cardiovasc Surg. 2005;130(5):1378.e1-1378.e10. 10.1016/j.jtcvs.2005.06.015. 16256792 10.1016/j.jtcvs.2005.06.015

[15] Diesch J, Zwick A, Garz AK, Palau A, Buschbeck M, Götze KS. A clinical-molecular update on azanucleoside-based therapy for the treatment of hematologic cancers. Clin Epigenetics. 2016;8(1):1–11. 10.1186/s13148-016-0237-y. 27330573 10.1186/s13148-016-0237-y PMC4915187

[16] Stein A, Platzbecker U, Cross M. How azanucleosides affect myeloid cell fate. Cells. 2022;11(16):2589. 10.3390/cells11162589. 36010665 10.3390/cells11162589 PMC9406747

[17] Yamada Y, Jackson-Grusby L, Linhart H, et al. Opposing effects of DNA hypomethylation on intestinal and liver carcinogenesis. Proc Natl Acad Sci USA. 2005;102(38):13580–5. 10.1073/pnas.0506612102. 16174748 10.1073/pnas.0506612102 PMC1224663

[18] Howard G, Eiges R, Gaudet F, Jaenisch R, Eden A. Activation and transposition of endogenous retroviral elements in hypomethylation induced tumors in mice. Oncogene. 2008;27(3):404–8. 10.1038/sj.onc.1210631. 17621273 10.1038/sj.onc.1210631

[19] Liouta G, Adamaki M, Tsintarakis A, et al. DNA methylation as a diagnostic, prognostic, and predictive biomarker in head and neck cancer. Int J Mol Sci. 2023;24(3):2996. 10.3390/ijms24032996. 36769317 10.3390/ijms24032996 PMC9917637

[20] Kim Y, Ko JY, Kong HK, et al. Hypomethylation of ATP1A1 is associated with poor prognosis and cancer progression in triple-negative breast cancer. Cancers. 2024. 10.3390/CANCERS16091666. 38730618 10.3390/cancers16091666 PMC11083557

[21] Hao X, Luo H, Krawczyk M, et al. DNA methylation markers for diagnosis and prognosis of common cancers. Proc Natl Acad Sci USA. 2017;114(28):7414–9. 10.1073/PNAS.1703577114/SUPPL_FILE/PNAS.1703577114.SAPP.PDF. 28652331 10.1073/pnas.1703577114 PMC5514741

[22] Agirre X, Vilas-Zornoza A, Jiménez-Velasco A, et al. Epigenetic silencing of the tumor suppressor microRNA Hsa-miR-124a regulates CDK6 expression and confers a poor prognosis in acute lymphoblastic leukemia. Cancer Res. 2009;69(10):4443–53. 10.1158/0008-5472.CAN-08-4025. 19435910 10.1158/0008-5472.CAN-08-4025

[23] Koelsche C, von Deimling A. Methylation classifiers: brain tumors, sarcomas, and what’s next. Genes Chromosom Cancer. 2022;61(6):346–55. 10.1002/GCC.23041. 35388566 10.1002/gcc.23041

[24] Song D, Yue L, Wu G, et al. Evaluation of promoter hypomethylation and expression of p73 as a diagnostic and prognostic biomarker in Wilms’ tumour. J Clin Pathol. 2016;69(1):12–8. 10.1136/JCLINPATH-2015-203150. 26184366 10.1136/jclinpath-2015-203150

[25] Brzezinski J, Choufani S, Romao R, et al. Clinically and biologically relevant subgroups of Wilms tumour defined by genomic and epigenomic analyses. Br J Cancer. 2021;124(2):437–46. 10.1038/s41416-020-01102-1. 33012783 10.1038/s41416-020-01102-1 PMC7853092

[26] Tang F, Lu Z, Lei H, et al. DNA methylation data-based classification and identification of prognostic signature of children with wilms tumor. Front Cell Dev Biol. 2021;9:1–13. 10.3389/fcell.2021.683242. 10.3389/fcell.2021.683242 PMC8740190 35004665

[27] Aryee MJ, Jaffe AE, Corrada-Bravo H, et al. Minfi: a flexible and comprehensive Bioconductor package for the analysis of Infinium DNA methylation microarrays. Bioinformatics. 2014;30(10):1363–9. 10.1093/bioinformatics/btu049. 24478339 10.1093/bioinformatics/btu049 PMC4016708

[28] Fortin JP, Triche TJ, Hansen KD. Preprocessing, normalization and integration of the Illumina HumanMethylationEPIC array with minfi. Bioinformatics. 2017;33(4):558–60. 10.1093/bioinformatics/btw691. 28035024 10.1093/bioinformatics/btw691 PMC5408810

[29] Zhou W, Laird PW, Shen H. Comprehensive characterization, annotation and innovative use of Infinium DNA methylation BeadChip probes. Nucleic Acids Res. 2017;45(4):e22. 10.1093/nar/gkw967. 27924034 10.1093/nar/gkw967 PMC5389466

[30] McCartney DL, Walker RM, Morris SW, McIntosh AM, Porteous DJ, Evans KL. Identification of polymorphic and off-target probe binding sites on the Illumina Infinium MethylationEPIC BeadChip. Genomics Data. 2016;9:22–4. 10.1016/j.gdata.2016.05.012. 27330998 10.1016/j.gdata.2016.05.012 PMC4909830

[31] Pidsley R, Zotenko E, Peters TJ, et al. Critical evaluation of the Illumina MethylationEPIC BeadChip microarray for whole-genome DNA methylation profiling. Genome Biol. 2016;17(1):1–17. 10.1186/s13059-016-1066-1. 27717381 10.1186/s13059-016-1066-1 PMC5055731

[32] Peters TJ, Buckley MJ, Chen Y, Smyth GK, Goodnow CC, Clark SJ. Calling differentially methylated regions from whole genome bisulphite sequencing with DMRcate. Nucleic Acids Res. 2021. 10.1093/nar/gkab637. 34320181 10.1093/nar/gkab637 PMC8565305

[33] Termuhlen AM, Tersak JM, Liu Q, et al. Twenty-five year follow-up of childhood wilms tumor: a report from the childhood cancer survivor study. Pediatr Blood Cancer. 2011;57:1210–6. 10.1002/pbc.23090. 21384541 10.1002/pbc.23090 PMC4634648

[34] Breslow NE, Lange JM, Friedman DL, et al. Secondary malignant neoplasms after Wilms tumor: an international collaborative study. Int J Cancer. 2010;127:657–66. 10.1002/ijc.25067. 19950224 10.1002/ijc.25067 PMC2878923

[35] Ko EY, Ritchey ML. Current management of Wilms’ tumor in children. J Pediatr Urol. 2009;5(1):56–65. 10.1016/j.jpurol.2008.08.007. 18845484 10.1016/j.jpurol.2008.08.007

[36] Dna EM, Cells HIC. DNA hypomethylation in cancer cells. Epigenomics. 2009;1(2):239–59. 10.2217/EPI.09.33. 20495664 10.2217/epi.09.33 PMC2873040

[37] Esteller M. CpG island hypermethylation and tumor suppressor genes: a booming present, a brighter future. Oncogene. 2002;21:5427–40. 10.1038/sj.onc.1205600. 12154405 10.1038/sj.onc.1205600

[38] Endo Y, Suzuki K, Kimura Y, et al. Genome-wide DNA hypomethylation drives a more invasive pancreatic cancer phenotype and has predictive occult distant metastasis and prognosis potential. Int J Oncol. 2022;60(6):1–12. 10.3892/ijo.2022.5351. 35419613 10.3892/ijo.2022.5351 PMC9015190

[39] Widschwendter M, Jiang G, Woods C, et al. DNA hypomethylation and ovarian cancer biology. Cancer Res. 2004;64(13):4472–80. 10.1158/0008-5472.CAN-04-0238. 15231656 10.1158/0008-5472.CAN-04-0238

[40] Barault L, Amatu A, Siravegna G, et al. Discovery of methylated circulating DNA biomarkers for comprehensive non-invasive monitoring of treatment response in metastatic colorectal cancer. Gut. 2018;67(11):1995–2005. 10.1136/gutjnl-2016-313372. 28982739 10.1136/gutjnl-2016-313372 PMC5897187

[41] Galanopoulos M, Tsoukalas N, Papanikolaou IS, Tolia M, Gazouli M, Mantzaris GJ. Abnormal DNA methylation as a cell-free circulating DNA biomarker for colorectal cancer detection: a review of literature. World J Gastrointest Oncol. 2017;9(4):142–52. 10.4251/wjgo.v9.i4.142. 28451061 10.4251/wjgo.v9.i4.142 PMC5390299

[42] García-Ortiz MV, Cano-Ramírez P, Toledano-Fonseca M, Aranda E, Rodríguez-Ariza A. Diagnosing and monitoring pancreatic cancer through cell-free DNA methylation: progress and prospects. Biomark Res. 2023;11(1):88. 10.1186/s40364-023-00528-y. 37798621 10.1186/s40364-023-00528-y PMC10552233

[43] Wang Y, Qian CY, Li XP, et al. Genome-scale long noncoding RNA expression pattern in squamous cell lung cancer. Sci Rep. 2015;5:1–11. 10.1038/srep11671. 10.1038/srep11671 PMC4498179 26159226

[44] Deng Y, Wu L, Ding Q, Yu H. AGXT2L1 is downregulated in carcinomas of the digestive system. Oncol Lett. 2020;20(2):1318–26. 10.3892/ol.2020.11645. 32724374 10.3892/ol.2020.11645 PMC7377163

[45] Shao T, Xie Y, Shi J, et al. Surveying lncRNA-lncRNA cooperations reveals dominant effect on tumor immunity cross cancers. Commun Biol. 2022;5(1):1–13. 10.1038/s42003-022-04249-0. 36463330 10.1038/s42003-022-04249-0 PMC9719535

[46] Zhang W, Shang S, Yang Y, et al. Identification of DNA methylation-driven genes by integrative analysis of DNA methylation and transcriptome data in pancreatic adenocarcinoma. Exp Ther Med. 2020;19:2963–72. 10.3892/etm.2020.8554. 32256782 10.3892/etm.2020.8554 PMC7086284

[47] Saindane M, Rallabandi HR, Park KS, et al. Prognostic significance of prostaglandin-endoperoxide synthase-2 expressions in human breast carcinoma: a multiomic approach. Cancer Inform. 2020. 10.1177/1176935120969696. 33223820 10.1177/1176935120969696 PMC7656875

[48] Murrell A, Heeson S, Reik W. Interaction between differentially methylated regions partitions the imprinted genes Igf2 and H19 into parent-specific chromatin loops. Nat Genet. 2004;36(8):889–93. 10.1038/ng1402. 15273689 10.1038/ng1402

[49] Murrell A, Ito Y, Verde G, et al. Distinct methylation changes at the IGF2-H19 locus in congenital growth disorders and cancer. PLoS ONE. 2008;3(3):1–7. 10.1371/journal.pone.0001849. 10.1371/journal.pone.0001849 PMC2268001 18365005

[50] Sparago A, Russo S, Cerrato F, et al. Mechanisms causing imprinting defects in familial Beckwith-Wiedemann syndrome with Wilms’ tumour. Hum Mol Genet. 2007;16(3):254–64. 10.1093/hmg/ddl448. 17158821 10.1093/hmg/ddl448

[51] Følling I, Wennerstrøm AB, Eide TJ, Nilsen HL. Phaeochromocytomas overexpress insulin transcript and produce insulin. Endocr Connect. 2021;10(8):815–24. 10.1530/EC-21-0269. 34170845 10.1530/EC-21-0269 PMC8346199

[52] Gao S, Lin Z, Li C, et al. LncINS-IGF2 promotes cell proliferation and migration by promoting G1/S transition in lung cancer. Technol Cancer Res Treat. 2019;18:1–10. 10.1177/1533033818823029. 10.1177/1533033818823029 PMC6374000 30803359

[53] Fleischer T, Tekpli X, Mathelier A, et al. DNA methylation at enhancers identifies distinct breast cancer lineages. Nat Commun. 2017. 10.1038/s41467-017-00510-x. 29123100 10.1038/s41467-017-00510-x PMC5680222

[54] Kreibich E, Kleinendorst R, Barzaghi G, Kaspar S, Krebs AR. Single-molecule footprinting identifies context-dependent regulation of enhancers by DNA methylation. Mol Cell. 2023;83(5):787-802.e9. 10.1016/j.molcel.2023.01.017. 36758546 10.1016/j.molcel.2023.01.017

[55] Sharifi-Zarchi A, Gerovska D, Adachi K, et al. DNA methylation regulates discrimination of enhancers from promoters through a H3K4me1-H3K4me3 seesaw mechanism. BMC Genomics. 2017;18(1):1–21. 10.1186/s12864-017-4353-7. 29233090 10.1186/s12864-017-4353-7 PMC5727985

