# Supplementary material for: Methylation changes and INS-IGF2 expression predict progression in early-stage Wilms tumor
Source: Clin Epigenetics. 2024 Nov 26;16:170. doi: 10.1186/s13148-024-01775-y (PMC11590261; doi:10.1186/s13148-024-01775-y)
Supplement: Supplementary file 1 — Additional file 1. [file 13148_2024_1775_MOESM1_ESM.docx]

**Supplementary Data**

**Supplementary Figure 1: UMAP clustering of CRT and RT samples across methylation probe subtypes.**

**

**
